# Afebrile Bacteremia in Adult Emergency Department Patients with Liver Cirrhosis: Clinical Characteristics and Outcomes

**DOI:** 10.1038/s41598-020-64644-7

**Published:** 2020-05-06

**Authors:** Hung-Yu Chen, Yin-Chou Hsu

**Affiliations:** Department of Emergency Medicine, E-Da Hospital, I-Shou University, Kaohsiung, Taiwan

**Keywords:** Diseases, Gastroenterology, Medical research

## Abstract

Cirrhotic patients with bacteremia are at an increased risk of organ failure and mortality. In addition, they can develop serious infection without fever because of their impaired immune response. Our study aimed to investigate the clinical characteristics and outcomes in afebrile bacteremic patients with liver cirrhosis. A single-center, retrospective cohort study was performed on adult patients who visited the emergency department from January 2015 to December 2018. All patients with bacteremia and diagnosis of liver cirrhosis were enrolled and classified as either afebrile or febrile. In total, 104 bacteremic patients with liver cirrhosis (afebrile: 55 patients and, febrile: 49) were included in the study. Compared with the febrile group, patients in the afebrile group showed a significantly higher rate of inappropriate antibiotics administration (43.6% vs. 20.4%, *p* = 0.01). They were also at an increased risk of 30-day mortality (40% vs. 18.4%, *p* = 0.02), intensive care unit transfer (38.2% vs. 18.4%, *p* = 0.03) and endotracheal intubation (27.3% vs. 10.2%, *p* = 0.03). The afebrile state was also an independent risk factor associated with 30-day mortality in cirrhotic patients with bacteremia. Clinicians should perform a prudent evaluation in cirrhotic patients and carefully monitor for possible signs of serious infection even in the absence of fever.

## Introduction

Patients with liver cirrhosis are prone to develop infection because of their cirrhosis-associated immune dysfunction, increased intestinal mucosa permeability and decreased hepatic bacteria filtration^1^. Among these, bacteremia is a serious and systemic infectious disease requiring aggressive treatment and investigation^2^. Compared to non-cirrhotic patients, bacteremia in cirrhotic patients shows significantly higher mortality and morbidity risk and prolonged hospitalization^3–5^.

Patients with fever accompanied by altered mental status and hypotension, may help clinicians to diagnose bacteremia^6^. Clinicians frequently rely on the presence of fever to initiate infection workup^7^; however, fever is a complex and non-specific host defense response against infection, and might be absent in bacteremic syndrome^8^. Afebrile bacteremic patients often have atypical clinical manifestations, such as lethargy or confusion^9^, leading to decreased survival and poorer prognosis^10^.

It is well known that patients with cirrhosis and bacterial infection frequently present with atypical manifestation, such as normothermia^11,12^; which makes the traditional Systemic Inflammatory Response Syndrome (SIRS) criteria inaccurate in the identification of patients with infection^11,13^. It has been proposed that scoring systems focused on organ dysfunction, such as quick Sequential Organ Failure Assessment (qSOFA) score or Chronic Liver Failure Sequential Organ Failure Assessment (CLIF-SOFA) score have better prediction ability in sepsis and prognosis stratification in patients with cirrhosis^13^. Despite this, previous studies regarding bacteremia in cirrhotic patients mainly focused on the severity of cirrhosis, bacteriology, source of infection, and presence of drug-resistant organisms; none of them addressed the issue of body temperature, which could influence the decision of treatment initiation^14–16^. Our study aimed to investigate the prevalence, clinical characteristics, and prognosis in afebrile bacteremic patients with liver cirrhosis.

## Methods

### Study design

A retrospective cohort study was conducted at a tertiary referral medical center with approximately 60,000 emergency department (ED) visits per year. We enrolled all adult patients (age ≥18 years) who visited the ED from January 1, 2015 to December 31, 2018 and fulfilled both criteria for further analysis: ED diagnostic code for liver cirrhosis (ICD10: K74.3, K74.4, K74.5, K74.60, K74.69, K71.7, K70.30, K70.31, K71.7); and positive blood culture results in ED. Patients with fungemia, contaminated blood samples, transferred from other medical facilities (including prior antibiotics use), and immunosuppressant use were excluded. During the ED course, physicians would collect blood samples from the patients for microbial culture and commence antimicrobial therapy accordingly if any of these criteria fulfilled: 1. Patients presented with a clinical manifestation of SIRS (e.g. fever, tachycardia); 2. Patients had evidence of infection by diagnostic test (e.g. chest radiograph, urinalysis) regardless of SIRS score; 3. Patients developed symptoms of acute on chronic liver failure (ACLF), such as jaundice, coagulopathy, hepatic encephalopathy, etc. Usually within 2–3 days after blood sample collection, the laboratory would notify attending physicians by telephone or message if they detected bacterial growth in incubated blood culture bottles; the species identification and the *in vitro* susceptibility were determined after 5–7 days of incubation. True bacteremia was defined as blood culture of ≥2 sets collected from separate sites yielding the same bacteria, or 1 set of blood culture yielding pathogen corresponding to the patient’s clinical manifestations. The study was carried out in accordance with the principles of the Declaration of Helsinki, and the protocol was approved by the local Institutional Review Board of E-DA hospital (EMRP-108–003). The requirement of informed consent was waived due to the retrospective observational nature of the study.

### Data collection and definitions

We collected data on demographic characteristics, initial ED vital signs, pre-existing co-morbidities, laboratory results, microbiological data, source of bacteremia, and initial use of antimicrobial agents from the manual chart review and electronic medical records of all eligible patients. We used the quick Sepsis Related Organ Failure Assessment (qSOFA) (positive if at least 2 of these criteria were met: altered mental status, respiratory rate ≥ 22 breaths/min, systolic blood pressure ≤ 100 mmHg) and the Systemic Inflammatory Response Syndrome (SIRS) (positive if at least 2 of these criteria were met: body temperature <36 °C or >38 °C, heart rate >90 beats/min, respiratory rate >20 breaths/min, white blood cells <0.4 × 10^9^/L or >1.2 × 10^9^/ L or bandemia ≥10%) scores for infection severity stratification in the bacteremic patients^17^. We stratified cirrhotic patients according to the etiology of cirrhosis (alcoholic, viral hepatitis, or others) and severity (Child-Pugh scores and, model of end-stage liver disease [MELD] scores)^18^, as well as the calculated parameters of organ dysfunction(chronic liver failure-sequential organ failure assessment [CLIF-SOFA] scores, and ACLF grade) in patients with cirrhosis^19^. The source of bacteremia was classified as respiratory tract infection (new infiltrate demonstrated on chest radiograph in a patient with a clinically compatible syndrome), urinary tract infection (urinalysis revealed pyuria [>10 white blood cells/mm^3^ per high-power field] and bacteriuria [urinary pathogen of ≥10^5^ colony-forming units/mL]), spontaneous bacterial peritonitis (diagnostic paracentesis with polymorphonuclear leukocyte count of ≥ 250 cells/µL), skin or soft tissue infection, biliary tract infection, and primary bacteremia (source of unknown origin). The methods of source control (e.g. debridement for soft tissue infection, drainage of abscess for intra-abdominal infection) were recorded according to the medical records.

### Outcomes measurement

All eligible patients of this study were divided into febrile and afebrile groups for primary and secondary outcomes measurement. The afebrile state was defined as temperature <38 °C in the tympanic membrane during the patient’s ED course (without any antipyretic agent use); otherwise any episode of tympanic membrane >38 °C measured during the patient’s ED course was defined as the febrile state. The patients were empirically treated with antibiotics in accordance with the recommendations of the European Association for the Study of Liver (EASL)^12^ and local epidemiological data (e.g., ceftriaxone for spontaneous bacterial peritonitis, amoxicillin/clavulanic acid for soft tissue infection, and moxifloxacin for respiratory tract infection). The initial antimicrobial therapy was considered “appropriate” if both of the following criteria were met: the antimicrobial regimen was administered within 24 hours after blood sample collection^20,21^, and the pathogen was susceptible to the antimicrobial agent based on the result of an *in vitro* susceptibility test. If these criteria were unfulfilled, the therapy was considered “inappropriate”. The primary outcome in this study was the 30-day mortality rate, while the secondary outcomes included rate of intensive care unit (ICU) transfer, endotracheal intubation (i.e. respiratory failure), shock (defined as need of vasopressors to maintain hemodynamic stability despite adequate fluid administration during ED stay), and renal replacement therapy administrated within hospital stay (i.e. renal failure).

### Statistical analysis

The characteristics of the afebrile and febrile bacteremic patients with liver cirrhosis were recorded and compared. Data were presented as mean with standard deviation or medians with interquartile range for continuous variables, and numbers (%) for categorical variables. Two-sample *t*-test and Chi-square tests were used to compare the continuous and categorical variables, respectively. The Mann-Whitney test was used for continuous variables if data were not normally distributed. The 30-day survival curves of the study groups were created using the Kaplan-Meier survival analysis, and the means were compared using the log-rank test. Cox proportional hazards regression model was used to analyze the independent variables associated with 30-day survival. We incorporated all variables with *p*-value < 0.1 in the univariate analysis into the regression model. A two-tailed *p*-value < 0.05 was considered statistically significant. All the statistical analyses were performed using the Statistical Package for the Social Sciences version 22.0 software (SPSS Inc, Chicago, IL, USA).

### Ethics approval and consent to participate

This observational study was approved by the Institutional Review Board of EDa hospital (EMRP-108-003), the informed consent was waived due to the retrospective observational nature of the study.

## Results

From January 1, 2015 to December 31, 2018, we identified 2362 adult ED cirrhotic patients, of which 1823 had hospital admission. The most common reason for admission was an infectious disease (52.6%), followed by gastrointestinal bleeding (27.3%). Blood cultures were drawn from 937 cirrhotic patients, of which 169 had positive blood culture results. After excluding patients with contaminated blood samples (N = 15), insufficient laboratory data (N = 18), inter-facility transfer (N = 23), and immunosuppressant use (N = 9), 104 bacteremic patients with liver cirrhosis (afebrile: N = 55; febrile: N = 49) were finally enrolled for further analysis.

### Demographics and clinical characteristics

As shown in Table 1, there were no statistically significant differences in the demographic variables (age, sex) between the afebrile and febrile groups. Furthermore, there were no underlying pre-existing comorbidities significantly associated with afebrile bacteremia. Regarding laboratory results, there were no significant differences between the afebrile and febrile groups, except for a lower hemoglobin level in the afebrile group (9.4 ± 2.3 vs. 10.6 ± 2.2 g/dL, *p* = 0.01). The afebrile and febrile groups did not differ in proportion with respect to etiology or severity of cirrhosis, although more than half of the patients in both groups belonged to Child’s C classification (70.9% vs. 61.2%, respectively, *p* = 0.30), indicating poor liver function performance. The distribution of ACLF grades was also similar in both groups. In the sepsis scoring system, a significantly lower proportion of the afebrile group fulfilled the SIRS criteria (49.1% vs. 93.9%, *p* < 0.001), while both groups showed non-significantly different distribution in the qSOFA scoring system (Table 1).

**Table 1:** Baseline characteristics of bacteremic patients with liver cirrhosis (n = 104).

Characteristics	Afebrile group (n = 55)	Febrile group (n = 49)	*p* value
Age, y, mean ± SD	55.3 ± 12.3	56.6 ± 12.1	0.58
Male, n (%)	41 (74.5)	39 (79.6)	0.54
**Laboratory results**			
Hemoglobin, g/dL, mean ± SD	9.4 ± 2.3	10.6 ± 2.2	0.01^*^
Leukocyte, ×10^9^/L, median(IQR)	10.6 (5.0–15.5)	8.6 (5.4–12.3)	0.18
Platelet, ×10^9^/L, median(IQR)	78 (46–115)	73.(41–125.5)	0.7
INR, median(IQR)	1.5 (1.3–1.9)	1.5 (1.3–1.8)	0.88
Bilirubin, mg/dL, median(IQR)	4.3 (2.9–10.3)	5.2 (2.4–7.9)	0.98
Sodium, mmol/L, mean ± SD	130 ± 9	130 ± 5	0.97
Creatinine, mg/dL, median(IQR)	1.5 (1–2.8)	1.3 (1.1–1.8)	0.36
eGFR, ml/min/1.73 m^2^, mean ± SD	50 ± 29	55 ± 22	0.35
CRP, mg/dL, median(IQR)	2.4 (0.7–7.0)	2.1 (0.8–6.9)	0.96
**Comorbidities, n (%)**			
Diabetes Mellitus	19 (35)	21 (42.9)	0.38
Hypertension	18 (32.7)	18 (36.7)	0.67
Malignancy	14 (25.5)	14 (28.6)	0.72
Indwelling catheter, n (%)	3 (5.5)	0 (0)	0.25
**Etiology of cirrhosis, n (%)**			
Alcohol	32 (58.2)	28 (57.1)	0.92
Viral Hepatitis	19 (34.5)	15 (30.6)	0.67
Other	4 (7.3)	6 (12.2)	0.51
Child-Pugh Classification, n (%)			0.46
A	3 (5.5)	2 (4.1)	
B	13 (23.6)	17 (34.7)	
C	39 (70.9)	30 (61.2)	
MELD score, mean ± SD	25 ± 7	25 ± 7	0.69
CLIF-SOFA score, mean ± SD	8 ± 3	8 ± 3	0.68
ACLF grade, n (%)			0.10
0	35 (63.6)	39 (79.6)	
1	15 (27.3)	4 (8.2)	
2	4 (7.3)	5 (10.2)	
3	1 (1.8)	1 (2.0)	
qSOFA score, n (%)			0.23
Altered mental status	12 (21.8)	16 (32.7)	
Respiratory rate ≥ 22 /min	11 (20.0)	13 (26.5)	
Systolic blood pressure ≤ 100 mmHg	14 (25.5)	12 (24.5)	
0	29 (52.7)	18 (36.7)	
1	18 (32.7)	22 (44.9)	
2	5 (9.1)	8 (16.3)	
3	3 (5.5)	1 (2.0)	
SIRS, n (%)	27 (49.1)	46 (93.9)	<0.001^*^

Abbreviations: SD, standard deviation; IQR, interquartile range; INR, international normalized ratio;

eGFR, estimated glomerular filtration rate; CRP, C-reactive protein; MELD, Model for End-Stage Liver Disease, calculated by following equation: 3.8 ×log_e_(serum bilirubin [mg/dL]) + 11.2 × log_e_(INR) + 9.6 × log_e_(serum creatinine [mg/dL]) + 6.4; CLIF-SOFA, Chronic Liver Failure-Sequential Organ Failure Assessment, stratified according to the serum total bilirubin, serum creatinine, hepatic encephalopathy grade, INR, mean arterial pressure, the ratio of partial arterial oxygen pressure to fraction of inspired oxygen (PaO2/FiO2);ACLF, acute on chronic liver failure; qSOFA, quick Sepsis Related Organ Failure Assessment, positive if at least 2 of these criteria were met: altered mental status, respiratory rate ≥ 22 breaths/min, systolic blood pressure ≤ 100 mmHg; SIRS, systemic inflammatory response syndrome, positive if at least 2 of these criteria were met: body temperature <36 °C or >38 °C, heart rate >90 beats/min, respiratory rate >20 breaths/min, white blood cells <0.4 × 10^9^/L or >1.2 × 10^9^/ L or bandemia ≥10%.

### Microbiological data, source of infection, and source control

Both groups presented predominantly gram-negative pathogen infection, followed by gram-positive strains (Table 2). The distribution of causative microorganisms was similar in both groups. The most common pathogens were *Escherichia coli*, followed by *Klebsiella pneumoniae* and *Staphylococcus aureus*. Regarding the source of infection, spontaneous bacterial peritonitis was the most common type of infection, followed by primary bacteremia and urinary tract infection, although all of them showed non-significantly different distribution in both groups (Table 2). The proportion of source control did not differ significantly between the two groups.

**Table 2:** Microbiological distribution, infection source and source control of bacteremic patients with liver cirrhosis (n = 104).

	Afebrile group (n = 55)	Febrile group (n = 49)	*p* value
Gram-positive pathogen, n (%)	19 (34.5)	14 (28.6)	0.51
*Staphylococcus aureus*	5 (9.1)	2 (4.1)	
Group B Streptococcus	3 (5.5)	1 (2.0)	
Viridans streptococcus	1 (1.8)	3 (6.1)	
Others	10 (18.2)	8 (16.3)	
Gram-negative pathogen, n (%)	31 (56.4)	31 (63.3)	0.47
*Escherichia coli*	12 (21.8)	12 (24.5)	
*Klebsiella pneumoniae*	9 (16.4)	8 (16.3)	
*Aeromonas sobria*	3 (5.5)	1 (2.0)	
*Aeromonas hydrophila*	1 (1.8)	3 (6.1)	
Others	6 (10.9)	7 (14.3)	
Polymicrobial, n (%)	5 (9.1)	4 (8.2)	1.00
**Infection source, n (%)**			
Respiratory tract infection	4 (7.3)	3 (6.1)	1.00
Urinary tract infection	6 (10.9)	7 (14.3)	0.60
Spontaneous bacterial peritonitis	20 (36.4)	17 (34.7)	0.86
Biliary tract infection	3 (5.5)	6 (12.2)	0.30
Soft tissue infection	9 (16.4)	5 (10.2)	0.36
Primary bacteremia^a^	13 (23.6)	11 (22.4)	0.89
Source control^b^, n (%)	5 (9.1)	4 (8.2)	1.00

^a^ - source of unknown origin.

^b^ - measures used to eliminate the source of infection.

### Outcomes

**Figure 1:**
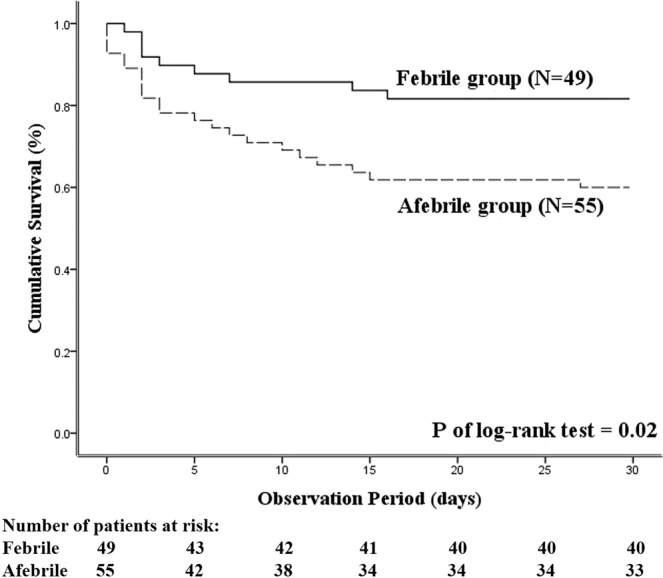
The Kaplan-Meier survival analysis for 30-day cumulative survival between the afebrile and febrile bacteremic patients with liver cirrhosis.

The mean initiation time of the antibiotic treatment was 3.5 hours in the patients (afebrile: 4.3 hours; febrile: 2.8 hours, *p* = 0.23). Eight patients, all of whom belonged to the febrile group, received delayed (more than 24 hours) antibiotic administration. Regarding the effectiveness of the antimicrobial agents, the following empirical drugs were ineffective in 31 patients (afebrile: 21; febrile: 10): third-generation cephalosporin (21/31), amoxicillin/clavulanic acid (6/31), and fluoroquinolones (2/31). Taken together, the rate of inappropriate antibiotic therapy was significantly higher in the afebrile group than in the febrile group (43.6% vs. 20.4%, *p* = 0.01) (Table 3).The overall 30-day mortality rate of our study cohort was 29.8% (31/104). The afebrile group showed a significantly higher 30-day mortality rate than the febrile group (40% vs. 18.4%, *p* = 0.02). The afebrile bacteremic patient group also had a higher rate of ICU transfer (38.2% vs. 18.4%, *p* = 0.03) and endotracheal intubation (27.3% vs. 10.2%, *p* = 0.03) (Table 3). There were no significant differences in septic shock development and renal replacement therapy administered between both groups. The Kaplan-Meier survival curves for 30-day survival comparing both groups are shown in Fig. 1. The 30-day cumulative survival probabilities in the afebrile and febrile groups were 60% and 81.6%, respectively (log-rank *p* = 0.02). Moreover, multivariate Cox proportional hazard regression analysis revealed that CLIF-SOFA score and afebrile state were independently associated with increased probabilities of 30-day mortality (Table 4).

**Table 3:** Outcome analysis of bacteremic patients with liver cirrhosis (n = 104).

Variables, n (%)	Afebrile group (n = 55)	Febrile group (n = 49)	*p* value
Inappropriate antibiotics use	24 (43.6)	10 (20.4)	0.01^*^
Effective antibiotics^a^	34 (61.8)	39 (79.6)	0.048^*^
Antibiotics within 24 hours	47 (85.5)	49 (100)	0.01^*^
Intensive care unit transfer	21 (38.2)	9 (18.4)	0.03^*^
Shock	26 (47.3)	23 (46.9)	0.97
Endotracheal intubation	15 (27.3)	5 (10.2)	0.03^*^
Renal replacement therapy	3 (5.5)	2 (4.1)	0.74
30-day mortality	22 (40)	9 (18.4)	0.02^*^

^a^-The isolated microorganism was sensitive to the selected antimicrobial agents based on an *in vitro* susceptibility test result.

**Table 4:** Multivariate Cox regression analysis of prognostic factors predicting 30-day mortality in bacteremic patients with liver cirrhosis (n = 104).

Variables, n (%)	Hazard Ratio	95% CI	*p* value
Male gender	0.71	0.17–2.97	0.64
Age (in year)	1.03	0.98–1.08	0.28
Child-Pugh score	1.08	0.75–1.56	0.66
MELD score	1.05	0.92–1.20	0.48
CLIF-SOFA score	1.68	1.17–2.41	<0.01^*^
Afebrile state	3.73	1.14–12.28	0.03^*^
Appropriate Antibiotics use	0.56	0.17–1.77	0.32

Abbreviations: CI, Confidence Interval.

## Discussion

In this ED-based single-center retrospective study, we investigated the prevalence, clinical characteristics and outcomes in afebrile bacteremic patients with liver cirrhosis. We demonstrated that although baseline characteristics were similar between the afebrile and febrile patients in our study cohort, delayed and improper selection of antimicrobial agents occurred more frequently in the afebrile group, which was at an increased risk of organ failure, including higher rate of ICU transfer and endotracheal intubation, further associated with higher mortality rate.

Previous studies considered afebrile bacteremia as a unique phenomenon in the elderly^9,22^ and immunocompromised patients^23^. These were not seen among our patients, probably due to their cirrhotic condition, which already caused dysregulated immune response and the absence of typical clinical manifestations compared with the general population^1^. Cirrhotic patients presented bacteremia more frequently than other comorbidities because of gut bacteria overgrowth and local immune defenses dysfunction^1^, further precipitated by polymorphonuclear leukocyte dysfunction and complement deficiency, leading to substantially high mortality rate (range 26–59%)^4,24^. Interestingly, cirrhosis itself has been recognized as a potential risk factor of afebrile bacteremia, further strengthening the distinct disease entity and treatment complexity^10^.

Our patients with cirrhosis were younger and predominantly male, and half of them had cirrhosis attributed to alcoholism, which was different from a recent epidemiological research on liver cirrhosis^25^. Although the prognosis and survival of patients between alcoholic and non-alcoholic cirrhosis were similar in previous studies^26,27^, alcoholic cirrhotic patients tended to have bacterial infections, less incidence of hepatocellular carcinoma formation, and more mortality events attributed to infectious disease^27^. Previous systemic reviews demonstrated that in hospitalized patients with decompensated cirrhosis related acute illness, median survival is <6 months with Child-Pugh score ≥10 or MELD score ≥18, which was seen in the majority of our cirrhotic patients^28^. These reports could explain why our sicker patient cohort was more susceptible to acute illness, especially infection events, resulting in worse outcome compared with other comorbidities^24^.

It is not surprising that the proportion of patients fulfilled the SIRS criteria in the afebrile group was far less than that of the febrile group because they were divided by body temperature, a determinant included in SIRS criteria. Nevertheless, SIRS criteria exhibited poor accuracy in diagnosing cirrhotic patients with a bacterial infection, including in-hospital mortality discrimination and septic shock development, ICU transfer or acute on chronic liver failure prediction^13^. Cirrhotic patients may have hypersplenism, use beta-blockers, and present leukopenia and bradycardia, thus showing a lack of the SIRS parameters^11,29^. Furthermore, reduced production of acute- phase proteins, such as C-reactive protein (CRP), in patients with decompensated liver cirrhosis, made them had a weaker severity prediction and stratification ability in response to infection^1,30^. In concurrence with these issues, more than half of our afebrile bacteremic patients presented absence of tachycardia or leukocytosis, neither with marked CRP elevation, which further lowered the warning level for clinicians, thereby dismissing infectious diseases^9,10^. Also, other scoring systems (such as Pitt bacteremia score) which rely on inflammatory parameters including the presentation of fever, revealed limited ability in bacteremia severity stratification in patients with cirrhosis^1^.

It had been proposed that markers of organ dysfunction rather than inflammatory variables have better prognosis impact and mortality prediction performance in cirrhotic patients with sepsis^13,19^. The CLIF-SOFA score, which incorporated six variables of organ dysfunction (kidney, liver, cerebral, lung, coagulation, circulatory), has been validated as a useful tool for short-term mortality prediction in cirrhotic patients with acute decompensation^19^. Acute-on-chronic liver failure (ACLF), which is characterized by acute deterioration of liver function with or without extra-hepatic organ failure, carries a significantly higher risk of mortality especially when accompanied by organ failure^12^. The bacterial infection is the common identifiable factor triggering ACLF, and both of them could result in liver function deterioration with multiple organ failure, and mortality in patients with cirrhosis^12,31^. In concert with this concept, the bacteremia events precipitated our fragile cirrhotic patients to both sepsis and ACLF progression, leading to mortality and other adverse outcomes. The independent 30-day mortality prediction ability of the CLIF-SOFA score in our regression model also strengthens the idea that the extent of organ dysfunction correlated better with the prognostic significance in patients with cirrhosis^19^.

Unlike other diseases that were more likely to have bacteremia with respiratory or urinary tract origin, cirrhotic patients tend to have spontaneous bacterial peritonitis as their primary infection source, which is consistent with our results^4,15,16^. The distribution of bacteremic isolates in our study cohort was similar to previous studies, predominantly presenting gram-negative pathogens including *Escherichia coli* and *Klebsiella pneumoniae* predominantly, suggesting that the gastrointestinal tract is the most common source of bacterial infection in cirrhotic patients^4,14–16,32^.

The timing and selection of antibiotics treatment differed significantly between our afebrile and febrile groups, with much higher rate of inappropriate use noted in the afebrile one. Prompt and appropriate antibiotics management is a crucial element in treating patients with sepsis^33^ as delayed antibiotics administration in patients with bacteremia has been recognized as an independent risk factor of mortality^1,12,32^. Interestingly, in contrast with the newest *Surviving Sepsis Campaign* guidelines that recommended the broad-spectrum antibiotics to be administrated within 1 hour for patients with sepsis and septic shock^33^, there were no unambiguous definitions regarding the “appropriate timing” of antibiotics administration in previous bacteremia studies, although most of them used 24 hours as the cut point^4,14,20,21^. The significantly higher rate of ineffective antibiotic administration in our patients (31/104, 29.8%) was unexpected, although the agents were prescribed in accordance with the international recommendations regarding the treatment of bacterial infection in patients with cirrhosis^12^. This was possibly because some of (13/31) the cases were classified as primary bacteremia that lack the typical signs of infection. Meanwhile, the high yield of multi-drug-resistant pathogens in our study is also consistent with the finding that the prevalence of multi-drug-resistant bacteria in cirrhotic patients has increased worldwide^12^. It was more prominent in the afebrile group who had blunted inflammatory response and showed absence of conventional inflammatory parameters, further complicating the identification of the infection source and choice of antimicrobial regimen^4,24^.

The 30-day mortality in our afebrile bacteremic group was substantially higher (40%), and was similar to a previous afebrile bacteremia study^10^. This could be attributed to their higher proportion of inappropriate antibiotics treatment, since timely and adequate antimicrobial therapy was still considered as an important prognostic factor in cirrhotic bacteremic patients, regardless of their comorbidities or infection severity^14,32,34^. In this study we demonstrated that body temperature is not a reliable marker for clinicians to differentiate infectious events in patients with cirrhosis, and could overlook the disease severity of the afebrile patients, thus delaying initiation of sepsis bundle, including antibiotics treatment, resulting in higher mortality risk. Another explanation for grave prognosis in the afebrile group is the consequence of their highly impaired systemic immune response to infection, predisposing serious complications and mortality, although this had not been validated by immunological assays^35^.

The lack of significant differences in the rate of septic shock between the two groups was unexpected, and probably because we only identified those patients needing vasoactive agents to maintain hemodynamic stability during the ED course as the shock condition, not taking account of the following ICU or ordinary ward course. Therefore, we may have missed a portion of patients who developed shock later. Also, we found no significant differences in the proportion of renal replacement therapy between the two groups, which could be because the majority of the patients (81/104, 77.9%) had no kidney failure defined in CLIF-SOFA scoring system (i.e., serum creatinine <2.0 mg/dl), hence only 6 of them received renal replacement therapy making the comparison less meaningful. Nevertheless, the analysis of cumulative survival probabilities and other organ failure parameters, including the rate of ICU transfer and respiratory failure, all indicated a far worse prognosis in afebrile patients.

There were several limitations in our study, including its monocentric and retrospective design. First, we did not calculate the detailed amount of fluid administration in bacteremic patients; although the source control was recorded and compared between the two groups, we didn’t take account of the actual timing of these interventions which could also influence the prognosis of patients with sepsis and septic shock^33^. Second, the different epidemiological data of our study cohort may limit the extrapolation ability of these results. Third, all data obtained from the manual chart reviews and electronic medical records made recall and selection bias inevitable. Fourth, our study failed to recognize patients who did not undergo blood culture tests in the ED, but developed bacteremia subsequently. Finally, some patients may have taken antipyretic agents before the ED visit, which may have influenced our stratification based on body temperature. Nonetheless, we defined the afebrile state as the absence of fever during the *entire* ED course, thus minimizing the effect of anti-pyretic use before the ED treatment.

In summary, afebrile bacteremic patients with liver cirrhosis conform a unique, but not a minority group. They have multifactorial immune system impairment and, lack of typical manifestations of infectious disease, which results in delayed diagnosis and inappropriate antimicrobial agent use. They carry an overwhelmingly higher rate of respiratory failure and, ICU transfer, further associated with a worse prognosis. Clinicians should pay more attention while treating cirrhotic patients, and rely not only on their body temperature or laboratory results; while the parameters of organ dysfunction such as the CLIF-SOFA score, have been validated as a more reliable prognostic factor in cirrhotic patients with bacteremia. They should always keep in mind the possibility of occult severe infection when unusual clinical manifestations are presented, such as lethargy, confusion, unexplained hypotension, or other symptoms of organ dysfunction. Only early recognition and prompt treatment can avoid deterioration of the patients and improve their outcomes.

## Data Availability

The datasets used and/or analyzed during the present study are available from the corresponding author on reasonable request.
